# IL-1β-Induced Downregulation of the Multifunctional PDZ Adaptor PDZK1 Is Attenuated by ERK Inhibition, RXRα, or PPARα Stimulation in Enterocytes

**DOI:** 10.3389/fphys.2017.00061

**Published:** 2017-02-07

**Authors:** Min Luo, Sunil Yeruva, Yongjian Liu, Giriprakash Chodisetti, Brigitte Riederer, Manoj B. Menon, Keisuke Tachibana, Takefumi Doi, Ursula E. Seidler

**Affiliations:** ^1^Department of Gastroenterology, Hepatology and Endocrinology, Hannover Medical SchoolHannover, Germany; ^2^Department of Infectious Diseases, the Second Affiliated Hospital of Chongqing Medical UniversityChongqing, China; ^3^Department of Endocrinology, the Second Affiliated Hospital of Chongqing Medical UniversityChongqing, China; ^4^Department of Biochemistry, Hannover Medical SchoolHannover, Germany; ^5^Laboratory of Molecular Medicine, Graduate School of Pharmaceutical Sciences, Osaka UniversityOsaka, Japan

**Keywords:** PDZ adaptor protein, intestinal inflammation, MAP kinase, electrolyte transport

## Abstract

**Background:** The PDZ adaptor protein PDZK1 modulates the membrane expression and function of a variety of intestinal receptors and ion/nutrient transporters. Its expression is strongly decreased in inflamed intestinal mucosa of mice and IBD patients.

**Aim and Methods:** We investigated whether the inflammation-associated PDZK1 downregulation is a direct consequence of proinflammatory cytokine release by treating intestinal Caco-2BBE cells with TNF-α, IFN-γ, and IL-1β, and analysing PDZK1 promotor activity, mRNA and protein expression.

**Results:** IL-1β was found to significantly decrease PDZK1 promoter activity, mRNA and protein expression in Caco-2BBE cells. A distal region of the hPDZK1 promoter was identified to be important for basal expression and IL-1β-responsiveness. This region harbors the retinoid acid response element RARE as well as binding sites for transcription factors involved in IL-β downstream signaling. ERK1/2 inhibition by the specific MEK1/2 inhibitors PD98059/U0126 significantly attenuated the IL-1β mediated downregulation of PDZK1, while NF-κB, p38 MAPK, and JNK inhibition did not. Expression of the nuclear receptors RXRα and PPARα was decreased in inflamed colonic-mucosa of ulcerative colitis patients and in IL-1β-treated Caco2-BBE cells. Moreover, the RAR/RXR ligand 9-cis retinoic acid and the PPARα-agonist GW7647 stimulated PDZK1 mRNA and protein expression and attenuated IL-1β-mediated inhibition.

**Conclusions:** The strong decrease in PDZK1 expression during intestinal inflammation may be in part a consequence of IL-1β-mediated RXRα and PPARα repression and can be attenuated by agonists for either nuclear receptor, or by ERK1/2 inhibition. The negative consequences of inflammation-induced PDZK1 downregulation on epithelial transport-function may thus be amenable to pharmacological therapy.

## Introduction

We recently reported that the expression of the PDZ-adaptor protein PDZK1 (NHERF3) is strongly reduced in inflamed intestine of ulcerative colitis patients and murine models of colitis (Lenzen et al., 2012; Yeruva et al., 2015). One consequence of the loss of PDZK1 expression is a defective regulation of the major intestinal sodium absorptive protein NHE3 (Cinar et al., 2007; Hillesheim et al., 2007; Zachos et al., 2009), but there are likely other defects, because PDZK1 binds to and regulates the function of an array of important intestinal transport proteins (Rossmann et al., 2005; Li et al., 2007; Sugiura et al., 2008, 2011; Moon et al., 2015). PDZK1 is an essential adaptor protein for a number of other important receptors, i.e., the hepatic HDL/LDL receptor SR-BI, and loss of its function leads to hypercholesterolemia and atherosclerosis (Kocher and Krieger, 2009). It also regulates the membrane expression and function of transporters in other organs, i.e., the urate transporter URAT (Endou and Anzai, 2008), the oxalate transporter CFEX (Thomson et al., 2005), and the multidrug resistance protein MRP4 in the kidney (Park et al., 2014). An inflammation-associated decrease of PDZK1 may thus be an important aspect of organ dysfunction in other inflammatory conditions in the body, and exploration of the molecular mechanisms of PDZK1 downregulation deserves further study.

One potential mechanism for the inflammation-associated decrease in PDZK1 in the intestine could be an alteration of the cellular composition of the intestinal mucosa or a change in the differentiation pattern of the enterocytes. Another potential mechanism could be a direct effect of proinflammatory mediators on PDZK1 synthesis in the epithelial cells. As these cytokines were found to be most consistently upregulated in intestinal tissues from IBD patients and an array of mouse models for intestinal inflammation (Dionne et al., 1997; Raddatz et al., 2005; Matsuda et al., 2009; Yeruva et al., 2010, 2015), we investigated the effect of TNF-α, IFN-γ [added because it may be necessary for inducing IL-1β and TNF-α receptors in intestinal epithelial cells and Caco2BBE cells (Panja et al., 1998; Wang et al., 2006)], and IL-1β on PDZK1 mRNA and protein expression as well as promoter activity in the human intestinal cell line Caco-2BBE. IL-1β was found to significantly decrease PDZK1 mRNA and protein expression, as well as promote NHE3 dysfunction. Studies were therefore performed toward the elucidation of the molecular mechanisms of this IL-1β-induced downregulation of PDZK1 and more importantly the question of its reversibility by pharmacological inhibition of the different IL-1β downstream signaling pathways.

## Materials and methods

### Reagents and antibodies

Human recombinant IL-1β, TNF-α, and IFN-γ were purchased from Sigma, Missouri, USA. Rabbit polyclonal PDZK1 antibody (PAB15564) was purchased from Abnova, Taipei, Taiwan. NF-κB inhibitor BAY11-7082 and p38MAPK inhibitor BIRB-796 were purchased from Calbiochem (Darmstadt, Germany) and Axon Medchem (Groningen, The Netherlands) respectively. JNK inhibitor SP600125, MEK1/2 inhibitor PD98059 and U0126 were purchased from Cell signaling Technologies, Frankfurt, Germany.

### PDZK 1 promoter constructs

PDZK1 promoter full length and different deletion constructs were explained in detail previously (Tachibana et al., 2008).

### Cell culture, seeding density, and cytokine treatment

Caco-2BBE cells were grown at 37°C in a humidified atmosphere containing 5% CO_2_ and 95% O_2_, in Dulbecco's Modified Eagle Medium containing 4.5 g·L^−1^ D-glucose and sodium pyruvate supplemented with 10% FCS, 50 units penicillin/streptomycin, and 1% non-essential amino acids. Huh-7 cells were grown in Dulbecco's Modified Eagle Medium containing 4.5 g·L^−1^ D-glucose and sodium pyruvate supplemented with 10% FCS, 50 units penicillin/ streptomycin. 0.25 × 10^6^ cells were plated in 6 well-plates and grown for 24 h, serum starved (0.5% FCS in medium) for 24 h and then treated with IL-1β (0.1, 1, or 10 ng·mL^−1^), TNF-α (up to 20 ng·mL^−1^) and IFN-γ (up to 30 ng·mL^−1^) for the times indicated in the respective figures. Concentration response curves for these cytokines in Caco-2BBE cells had been tested in a previous study (Yeruva et al., 2008), and were applied accordingly in this study. For each experiment, two (qPCR) or three (Western Analysis) individual dishes were studied for each experimental condition, and the results pooled for *n* = 1. Each experiment was repeated three times in different cell passages. *n* indicates the number of repeats in different cell passages. Due to the homogeneity of a cell line, and the duplicate or triplicate measurements within the same experiment, we believe that three repeats are acceptable for a statistically valid conclusion.

### Transient transfections and luciferase assays

3 × 10^4^ cells were seeded in 24 wells and grown overnight. Two hundred and fifty nanograms of plasmid per well was mixed with 10 ng of renilla luciferase plasmid and transfections were done using a Jet prime® polyplus transfection reagent from Peqlab (Erlangen, Germany) according manufacturer's protocol. Cells were serum starved overnight before adding cytokines and then treated with cytokines for the time periods indicated in the text. After the treatment cells were lysed in 1X passive lysis buffer (Promega) by shaking at room temperature for 15 min. Luciferase assay was performed as described previously (Menon et al., 2011).

### Patients selection

The details of the patients who provided the biopsies of UC patients are given in detail in a previous report (Yeruva et al., 2015).

### Inhibition of NF-κB and MAPKs pathways

For NF-κB and MAPK pathway inhibition experiments, Caco-2BBE cells were pretreated with a NF-κB inhibitor BAY11-7082 (10 μM), a p38MAPK inhibitor BIRB-796 (μM), a JNK inhibitor SP600125 (25 μM), MEK1/2 inhibitor PD98059 (30 μM), and U0126 (10 μM) for 1 h, followed by exposure to IL-1β (10 ng·mL^−1^) for 48 h. Cells were lysed for Western blot analysis as described below.

### 9-*cis* retinoic acid treatment experiments

For PDZK1 mRNA measurements, Caco-2BBE cells were pretreated for 30 mins with 9-*cis* retinoic acid (RA) or vehicle at a concentration of 1 μM and then treated with IL-1β for 3, 6, 12, and 24 h. For PDZK1 protein assessment, the cells were pretreated with 1 μM RA or vehicle for 30 min before addition of IL-1β (10 ng·mL^−1^) and the cells were harvested after 48 h.

### RNA isolation and real-time PCRs

RNA isolation from cells was done using Qiagen RNA isolation kit and real time PCRs were performed as explained previously (Yeruva et al., 2015).

### Immunoblot analysis

After the treatment, cells were lysed in lysis buffer (M-PER® Mammalian protein extraction reagent from Thermo scientific, Rockford, USA) and protein concentration was estimated with Bio-rad Bradford assay. Twenty to forty micrograms of total cellular proteins were separated on 8–10% SDS-poly acryl amide gels and transferred to polyvinylene difluoride membranes. Antibodies were diluted in TBST containing 5% non-fat dry milk and blots were incubated overnight at 4°C, washed with TBST and incubated with secondary antibodies conjugated to horseradish peroxidase, washed with TBST and then developed using enhanced chemiluminescence kit from GE health sciences.

### WST-1 cell viability assay

The reagent WST-1 was used to determine cell viability according to the manufacturer's instructions. In brief, Caco-2BBE cells were seeded at a density of 1 × 10^4^ cells in each well of a 96 well-plate and were grown and treated as stated in the section “Cell culture, seeding density, and cytokine treatment.” Cells were incubated with respective cytokines for 24 h. At the end of treatment, 10 μl WST-1 were added to each well and 1 h later absorbance was measured at 450 and 630 nm using the BioTek® Epoch Reader. No decrease in viability was detected during exposure of any of the tested cytokines or their combination (Supplementary Figure 5).

### Statistical analysis

Results are given as means ± *SEM* (Standard error of mean). We usually performed 3–4 independent experiments in different passages of cells, where individual data points were based on at least triplicates for each condition. Unpaired Student's two-tailed *t*-test were used for comparing between two groups. One or Two-way analysis of variance (ANOVA) with *post-hoc* analysis including Dunnett's Multiple Comparison Test and Bonferroni post-tests were used for multiple comparisons. All analyses were performed on GraphPad Prism version 6.0 and *P* < 0.05 were considered significant. ^*^P < 0.05, ^**^P < 0.01, ^***^P < 0.001.

## Results

### Interleukin-1β strongly down regulates PDZK1 mRNA and protein expression as well as PDZK1 promoter activity in Caco-2BBE cells in a concentration- and time-dependent manner

**Figure 1:**
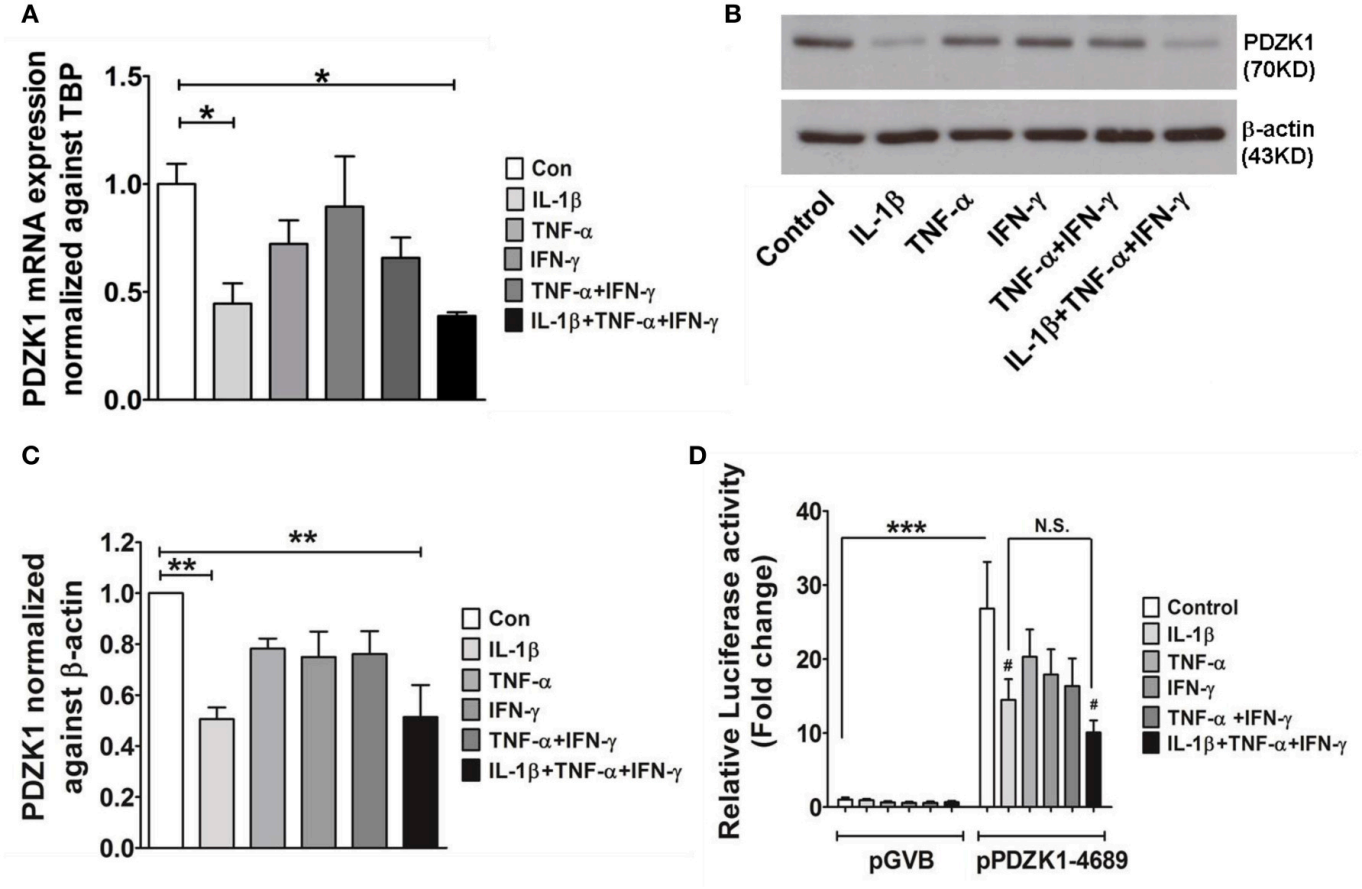
**Cytokine mediated regulation of PDZK1 in Caco-2BBE cells. (A)** PDZK1 mRNA expression in Caco-2BBE cells was analyzed by real-time PCRs after treatment with IL-1β (10 ng·mL^−1^), TNF-α (20 ng·mL^−1^), and IFN-γ (30 ng·mL^−1^) alone or in combination, data are represented as fold change in expression normalized to TBP. **(B)** PDZK1 protein expression was analyzed by Western blots using β-actin as a loading control. **(C)** Western quantification using image J software. Among the treated cytokines, IL-1β leads to more than a 50% decrease in PDZK1 mRNA and protein expression. Bar graphs are represented as mean ± SEM. ^*^indicates *P* < 0.05 and ^**^indicates *P* < 0.01 compared to control, *n* = 4 experimental repeats in different passages. **(D)** PDZK1 promoter activity was normalized to the pGVB (vector backbone) transfected control cells and shown as relative fold change. Among the different cytokine treatments IL-1β alone had strong inhibition of PDZK1 promoter activity. Bar graphs are represented as mean ± SEM. ^***^P < 0.001 compared to vector back bone and ^#^*P* < 0.001 compared to control. *n* = 4 experimental repeats done in triplicates for each condition.

Caco-2BBE cells were treated with IL-1β (10 ng·mL^−1^), TNF-α (20 ng·mL^−1^), and IFN-γ (30 ng·mL^−1^), alone or in combination and effects on PDZK1 mRNA and protein expression were assessed after 24 h of incubation. IL-1β had the strongest inhibitory effect on PDZK1 mRNA expression (Figure 1A), and was the only tested cytokine which significantly decreased protein expression at that time point (Figures 1B,C). The combination of all three cytokines did not show a stronger inhibition of PDZK1 gene expression than IL-1β alone and the effect of TNF-α and/or IFN-γ on PDZK1 mRNA expression did not reach the level of significance at 24 h exposure time (Figure 1A). The full length PDZK1 promoter containing luciferase reporter plasmid (pPDZK1-4689) displayed robust basal promoter activity in Caco-2BBE cells (26.8 ± 6.3-fold increase of luciferase activity compared to vector backbone expression), which was significantly decreased in the presence of 10 ng·mL^−1^ IL-1β (Figure 1D). Treatment of Caco-2BBE cells with either TNF-α or IFN-γ did not result in significant inhibition, and the combination or all the three cytokines together did not cause a stronger inhibition than IL-1β alone (14.5 ± 2.8-fold with IL-1β vs. 10.0 ± 1.7-fold with the cytomix, ns; Figure 1D).

**Figure 2:**
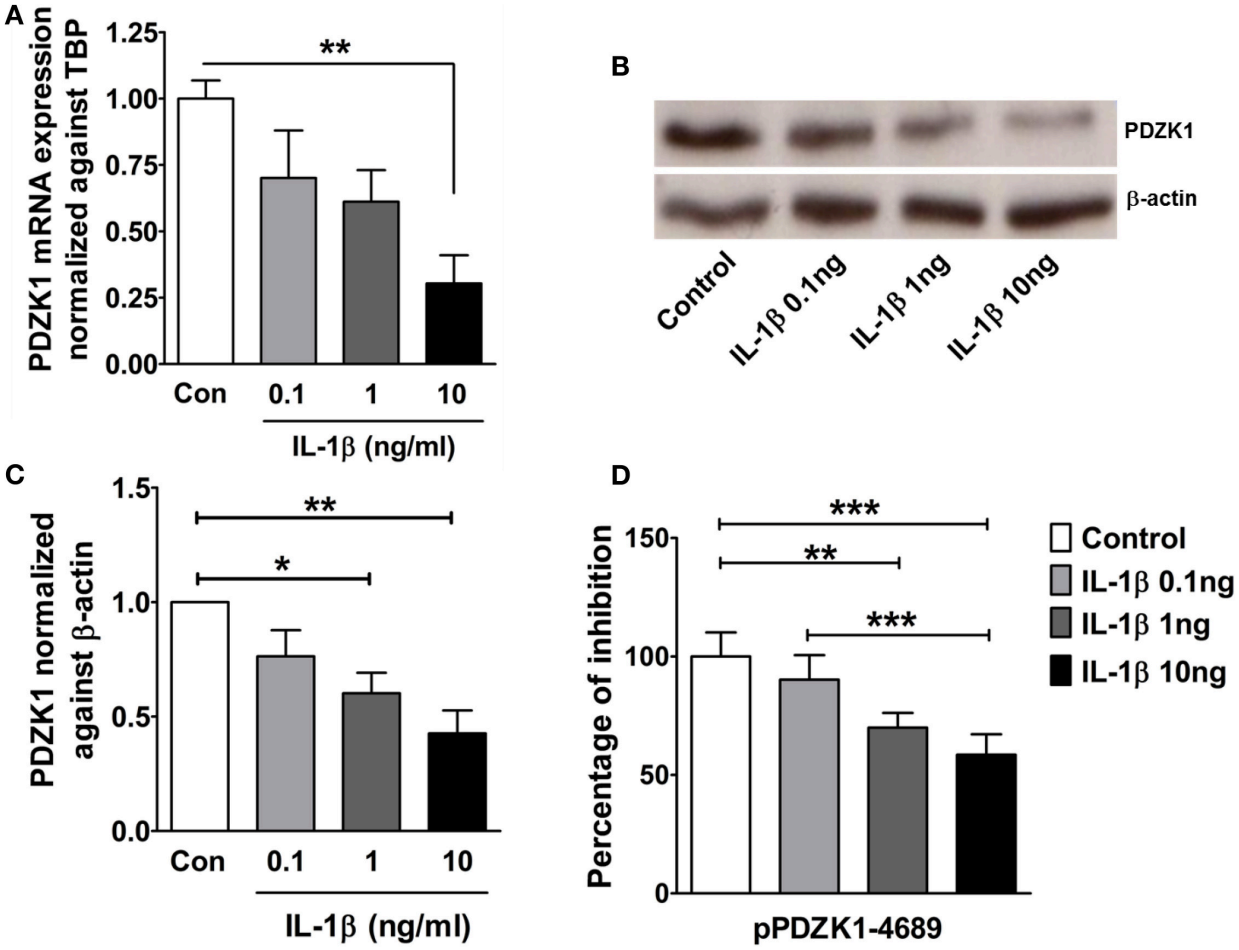
**Dose dependent downregulation of PDZK1 mRNA and protein expression as well as PDZK1 promoter activity in IL-1β treated Caco-2BBE cells. (A)** PDZK1 mRNA expression in Caco-2BBE cells was analyzed by real-time PCRs after treatment with increased concentration of IL-1β (0.1, 1, 10 ng·mL^−1^), data are represented as fold change in expression normalized to TBP. **(B)** PDZK1 protein expression was analyzed by Western blots using β-actin as a loading control. **(C)** Western quantification using image J software. IL-1β downregulated PDZK1 protein expression in a dose dependent manner. Bar graphs are represented as mean ± SEM. ^*^indicates *P* < 0.05 and ^**^indicates *P* < 0.01 compared to control, *n* = 4 experimental repeats in different passages. **(D)** Dose dependent inhibition of PDZK1 promoter activity by IL-1β in Caco-2BBe cells. Bar graphs are represented as mean ± SEM. ^**^indicates *P* < 0.01 and ^***^indicates *P* < 0.001. *n* = 3 experimental repeats done in triplicates for each condition.

**Figure 3:**
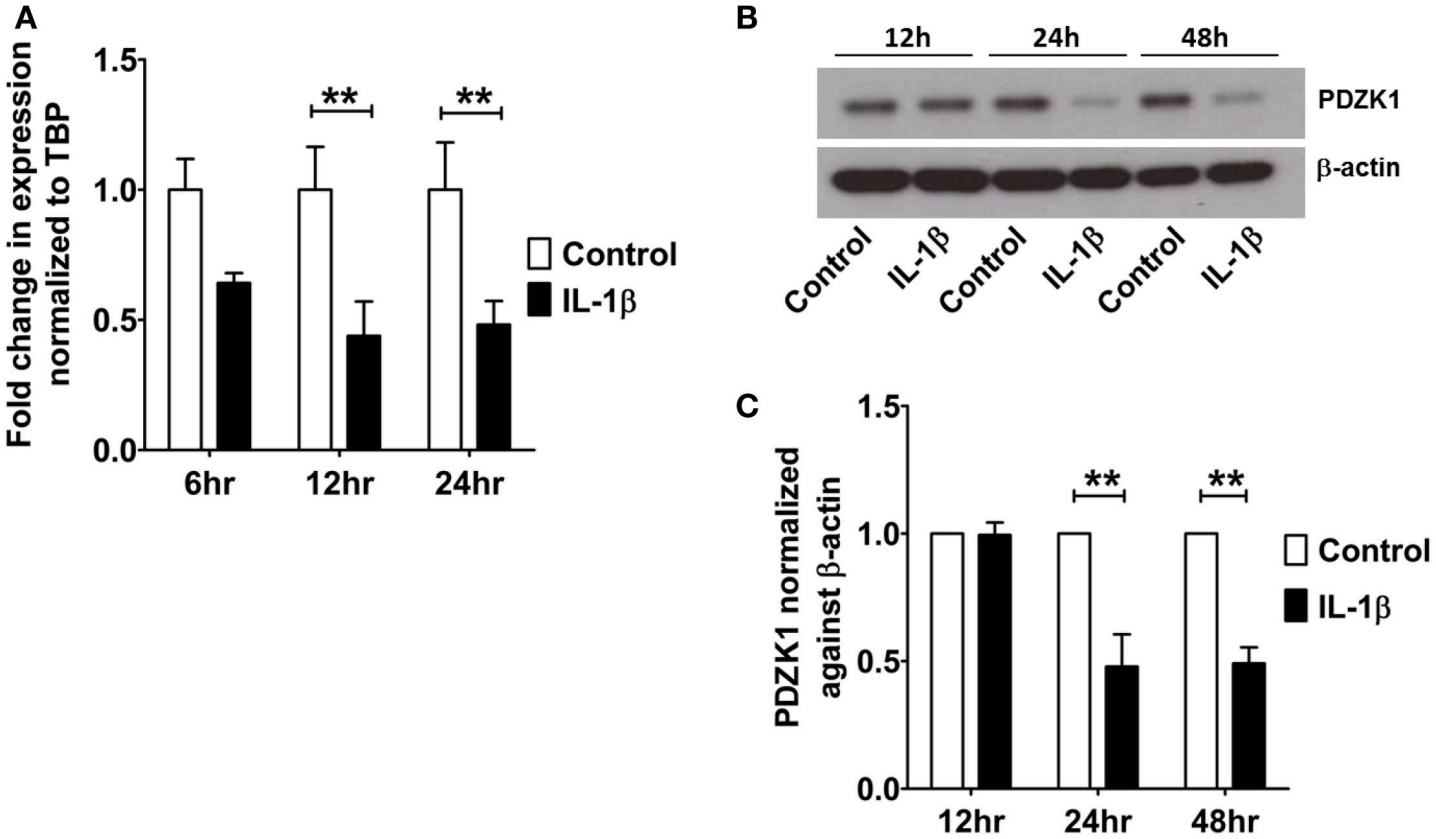
**Time kinetics of IL-1β mediated PDZK1 downregulation in Caco-2BBE cells. (A)** PDZK1 mRNA expression was analyzed by real-time PCRs in Caco-2BBE cells in the presence of 10 ng·mL^−1^ of IL-1β at different time points (6, 12, and 24 h) and data are represented as fold change in expression normalized to TBP. **(B,C)** PDZK1 protein expression was analyzed by Western blots in Caco-2BBE cells in the presence of 10 ng·mL^−1^ of IL-1β at different timepoints (12, 24, and 48 h). Bar graphs are represented as mean ± SEM. ^**^indicates *P* < 0.01 compared to control, *n* = 4 experimental repeats in different passages, done in triplicates for each condition.

The effect of IL-1β on PDZK1 mRNA (Figure 2A) and protein expression (Figures 2B,C) as well as PDZK1 promoter activity (Figure 2D) was concentration-dependent, with a maximal inhibition of PDZK1 mRNA expression to 30.3 ± 10.6% of the value of vehicle treated controls at 10 ng·mL^−1^ IL-1β, and of PDZK1 protein expression to 42.6 ± 10% at 10 ng·mL^−1^ IL-1β. Treatment of Caco-2BBE cells with IL-1β (one single addition at time 0) for 6, 12, and 24 h revealed that PDZK1 mRNA expression was significantly downregulated within 12 h after 10 ng·mL^−1^ IL-1β addition to 43.8 ± 13.2% of vehicle-treated controls, and remained downregulated after 24 h (Figure 3A). The effect of IL-1β on PDZK1 protein expression was not significant at 12 h, whereas by 24 h IL-1β had a strong inhibitory effect to 52.2 ± 12.6% and this inhibitory effect was persistent at 48 h (50.9 ± 6.3% of vehicle-treated control value, *n* = 3; Figures 3B,C). We also studied the effect of the same cytokines on PDZK1 expression in the hepatoma cell line Huh-7, which also express PDZK1. Treatment with IL-1β led to a significant decrease of PDZK1 protein expression in Huh-7 cells (45.8 ± 2.2% of controls; Supplementary Figure 1), suggesting that a more general signaling pathway, rather than enterocyte-specific mediators are involved in PDZK1 downregulation.

### The 5′ end of the PDZK1 promoter is important for basal activity and IL-1β downregulation of promoter activity

**Figure 4:**
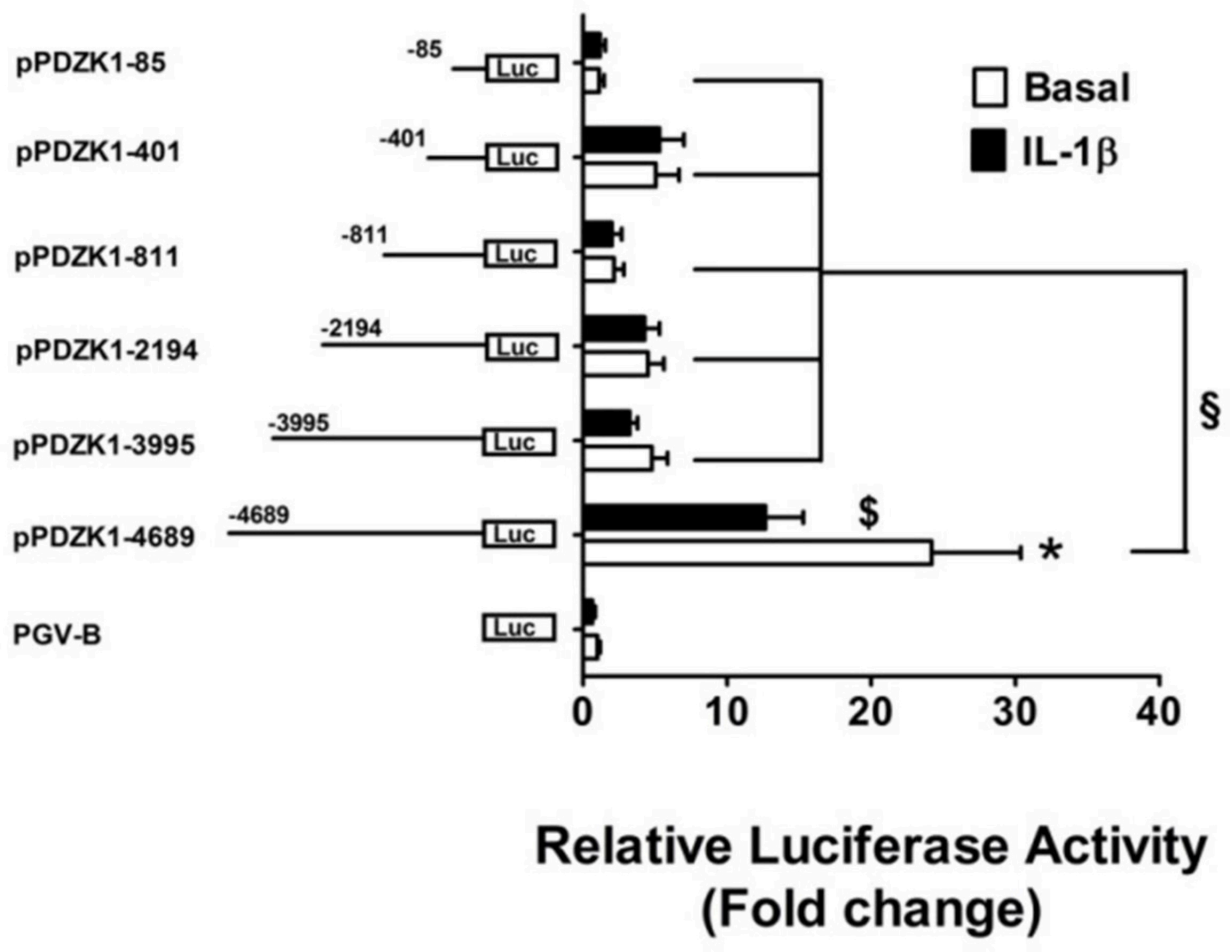
**Identification PDZK1 promoter region important for basal expression and IL-1β responsiveness**. Caco-2BBE cells were transiently transfected with a series of 5′-deletion PDZK1 promoter constructs and treated with and without IL-1β (10 ng·mL^−1^) for 24 h. Cells were harvested and luciferase assays were performed as described in materials and methods. IL-1β responsiveness and basal promoter activity was abolished after the deletion of the PDZK1 promoter region between 5′ 4689 and 3995 bp. Bar graphs are represented as mean ± SEM. ^*^indicates *P* < 0.001 pGVB compared to pPDZK1-4689, $indicates *P* < 0.001 IL-1β compared basal and §indicates *P* < 0.001, pPDZK1-4689 compared to the rest of deletion constructs. *n* = 3 experimental repeats, done in triplicates for each condition.

Transient transfections of PDZK1 promoter 5′-deletion reporter constructs in Caco-2BBE cells revealed that deletion of base pairs –4689 to –3995 led to a strong decrease of basal PDZK1 promoter activity by 80.2% of that of the full length promoter, and a significant loss of IL-1β mediated inhibition (Figure 4). This region harbors numerous transcription factor binding sites, which in part are known downstream targets for IL-1β-receptor-activated kinases (see next paragraph), as well as the retinoic acid response element (RARE; Supplementary Figure 2).

### Effect of blocking IL-1β receptor downstream signaling pathways on the inhibition of PDZK1 expression by IL-1β

**Figure 5:**
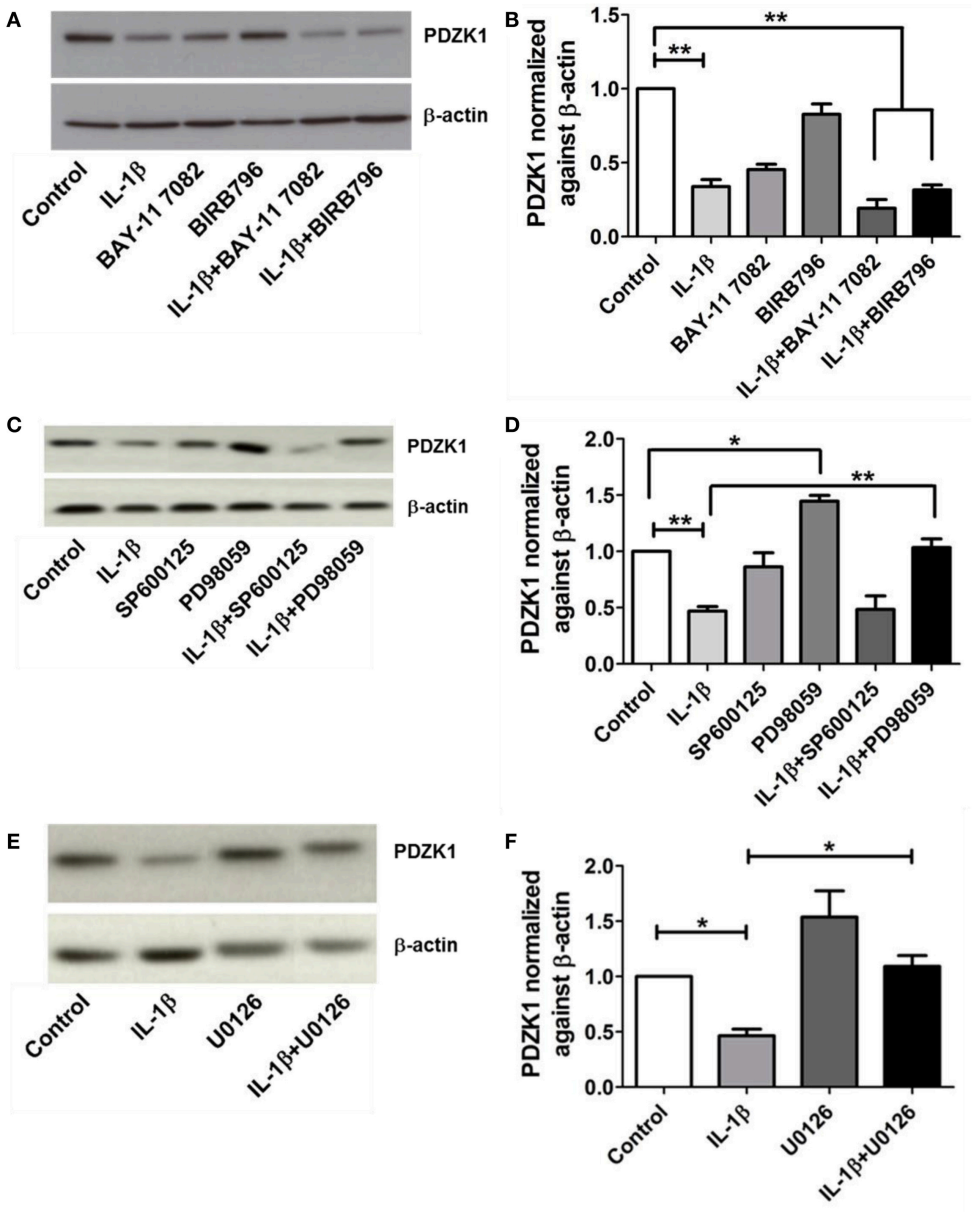
**Effects of NF-kB, p38-MAPK, JNK, and ERK 1/2 inhibitors on PDZK1 protein expression**. **(A)**. Caco-2BBE cells were pre-treated for 1 h with BAY11-7082 (10 μM) and BIRB-796 (1 μM) alone and in combination and then treated for 48 h with IL-1β (10 ng·mL^−1^). PDZK1 protein expression was analyzed by Western blots using β-actin as a loading control. **(B)** Western quantification using image J software. Both the inhibitors either used alone or in combination couldn't reverse the effect of IL-1β on PDZK1 protein expression. **(C)** However, pre-treatment of Caco-2BBe cells with JNK1-3 (25 μM of SP600125) and ERK1/2 (30 μM of PD98059) signal pathways inhibitor **(C,D)** revealed the involvement of ERK1/2 pathway in IL-1β mediated PDZK1 downregulation. PDZK1 protein expression was analyzed by Western blots using β-actin as a loading control. **(D)** Western quantification using image J software. JNK1-3 inhibitor SP600125 couldn't diminish the effect of IL-1β on PDZK1 protein expression. ERK1/2 inhibition by PD98059 stimulated PDZK1 expression and attenuated the IL-1β-induced PDZK1 downregulation. **(E)** Caco-2BBE cells were pretreated for 1 h with ERK 1/2 inhibitor U0126 (10 μM) before IL-1β (10 ng·mL^−1^) treatment for 48 h. PDZK1 protein expression was analyzed by Western blots using β-actin as a loading control. **(F)** Western quantification using image J software. U0126 could reverse the effect of IL-1β on PDZK1 protein expression. Bar graphs are represented as mean ± SEM. ^*^indicates *P* < 0.05 and ^**^indicates *P* < 0.01. *n* = 3 experimental repeats in different cell passages, triplicate cell dishes for each experimental condition.

IL-1β receptor activation exerts its cellular effects via several downstream pathways, including NF-κB, p38MAPK, ERK1/2, and JNK pathways (Weber et al., 2010). The most specific inhibitors for the respective signaling pathways were selected (Davies et al., 2000; Bain et al., 2003, 2007) and tested in various concentrations for their ability to attenuate the effect of IL-1β on PDZK1 protein expression. Pre-treatment with 10 μM of the NF-κB inhibitor BAY11-7082, 1 μM of the p38MAP kinase inhibitor BIRB-796 (Figures 5A,B) and 25 μM of the JNK1-3 inhibitor SP600125 did not significantly diminish the effect of IL-1β on PDZK1 protein expression (Figures 5C,D). In contrast, inhibition of the ERK1/2-cascade by two different MEK-inhibitors, namely PD98059 (Figures 5C,D) and U0126 (Figures 5E,F), both significantly stimulated PDZK1 expression to 144.6 ± 5.1% of vehicle-treated control for PD98059, and 153.7 ± 13.8% of vehicle-treated control for U0126 and significantly attenuated the IL-1β-induced PDZK1 downregulation (53.2 ± 4.1% inhibition in the absence vs. 28.1 ± 7.4% in the presence of PD98059, *p* < 0.05, *n* = 3, and 53.6 ± 5.3% inhibition in the absence vs. 29.6 ± 3.1% in the presence of 10 μM U0126, *p* < 0.05, *n* = 3).

### Effect of retinoic acid on PDZK1 expression

**Figure 6:**
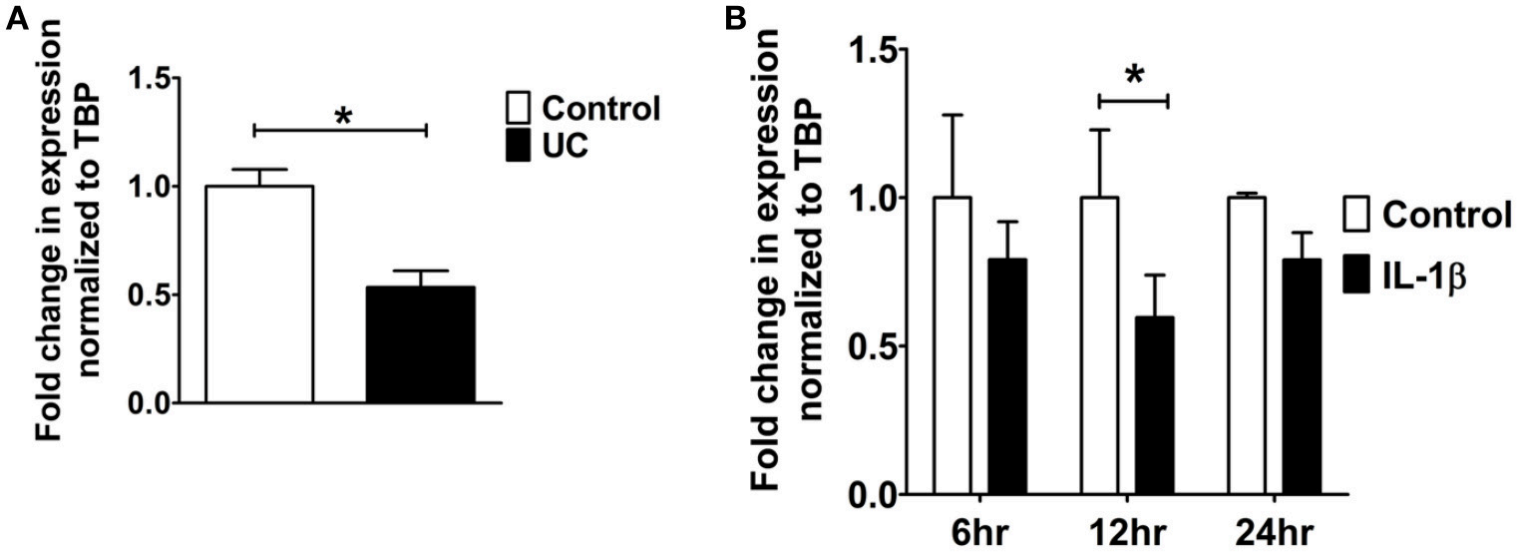
**RXR-α mRNA expression analysis from UC patients and Caco-2BBE cells. (A)** RXR-α mRNA expression in biopsies of ulcerative colitis patients which were used in our previous study. A geometrical mean of two housekeeping genes (TBP and β-actin) were used as normalization controls as explained in our previous study (Yeruva et al., 2010). **(B)** RXR-α mRNA expression in Caco-2BBE cells treated with and without IL-1β for different time points (6, 12, and 24 h) as depicted in the bar graph. IL-1β significantly downregulated RXR-α mRNA levels by 12 h. *n* = 3 experimental repeats done in duplicates for each condition. Bar graphs are represented as mean ± SEM. ^*^indicates *P* < 0.05 compared to control.

**Figure 7:**
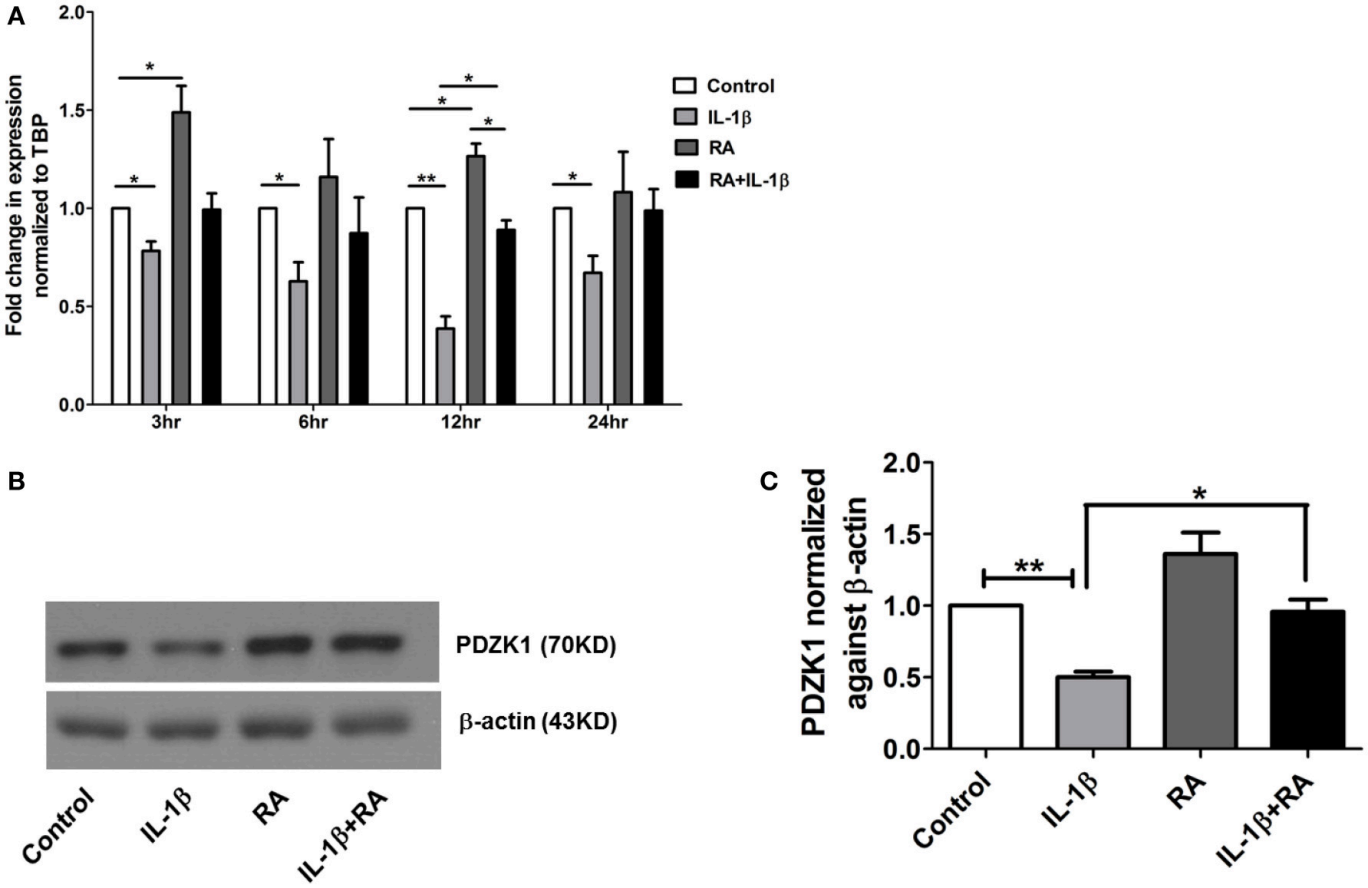
**9-***cis*** retinoic acid (RA) attenuated IL-1β induced PDZK1 protein downregulation. (A)** Caco-2BBE cells were pre-treated for 1 h with 9-*cis* retinoic acid (RA) at a concentration of 1 μM and before IL-1β (10 ng·mL^−1^) for treatment at different time pint (3, 6, 12, and 24 h). Cells were harvested after the treatment for RXR-α mRNA analysis. Treatment with RA clearly abrogated the IL-1β mediated PDZK1 down regulation. *n* = 3 experimental repeats done in duplicates (different cell dishes) for each condition. Bar graphs are represented as mean ± SEM. ^*^indicates *p* < 0.05 compared to control. **(B)** Caco-2BBe cells were pre-treated with RA (1 μM) and then treated with IL-1β (10 ng·mL^−1^) for 48 h before lysing the cells for protein expression study. Western blot analysis of PDZK1 protein expression and **(C)** Image J quantification of the blots normalized to β-actin expression. Treatment with RA clearly abrogated the IL-1β mediated PDZK1 down regulation in Caco-2BBE cells. Bar graphs are represented as mean ± SEM. *n* = 3 experimental repeats in different passages, done in triplicates for each condition. Bar graphs are represented as mean ± SEM. ^*^indicates *P* < 0.05 and ^**^indicates *P* < 0.01.

9 *cis*-retinoic acid (RA), a ligand for both RAR and RXR receptors, increased PDZK1 mRNA expression levels in Caco-2BBE cells 3 h after application (**Figure 7A**). qPCR analysis revealed that RXR-α mRNA expression was significantly decreased in intestinal mucosa of patients with active ulcerative colitis (Figure 6A). It was also significantly decreased 12 h after IL-1β addition to Caco-2BBE cells, with restoration to preincubation levels after 24 h (Figure 6B). This suggests that an IL-1β-mediated RXR-α depression may be involved in inflammation-associated PDZK1 downregulation. We tested whether the inhibitory effect of IL-1β on PDZK1 protein expression was, in part, reversible by simultaneous RA application. RA resulted in a significant increase of PDZK1 mRNA at 12 h (Figure 7A), and protein expression at 24 h (Figures 7B,C) both in the absence and presence of IL-1β. PDZK1 mRNA and protein expression was restored to control levels after co-administration of both substances. Figure 7A displays the different time dynamics of the effect of RA and IL-1β on PDZK1 mRNA expression, with a rapid but less sustained action of RA than of IL-1β. Twenty-four hours after the addition of both substances, the percentage-inhibitory effect of IL-1β on PDZK1 expression was 32.9 ± 8.7% in the absence and 9.4 ± 29.3% in the presence of RA at the mRNA level, and was 49.9 ± 3.8% in the absence and 28.5 ± 4.0% in the presence of RA at the protein level (Figures 7A–C).

### Effect of the PPARα agonist GW7647 on PDZK1 expression

**Figure 8:**
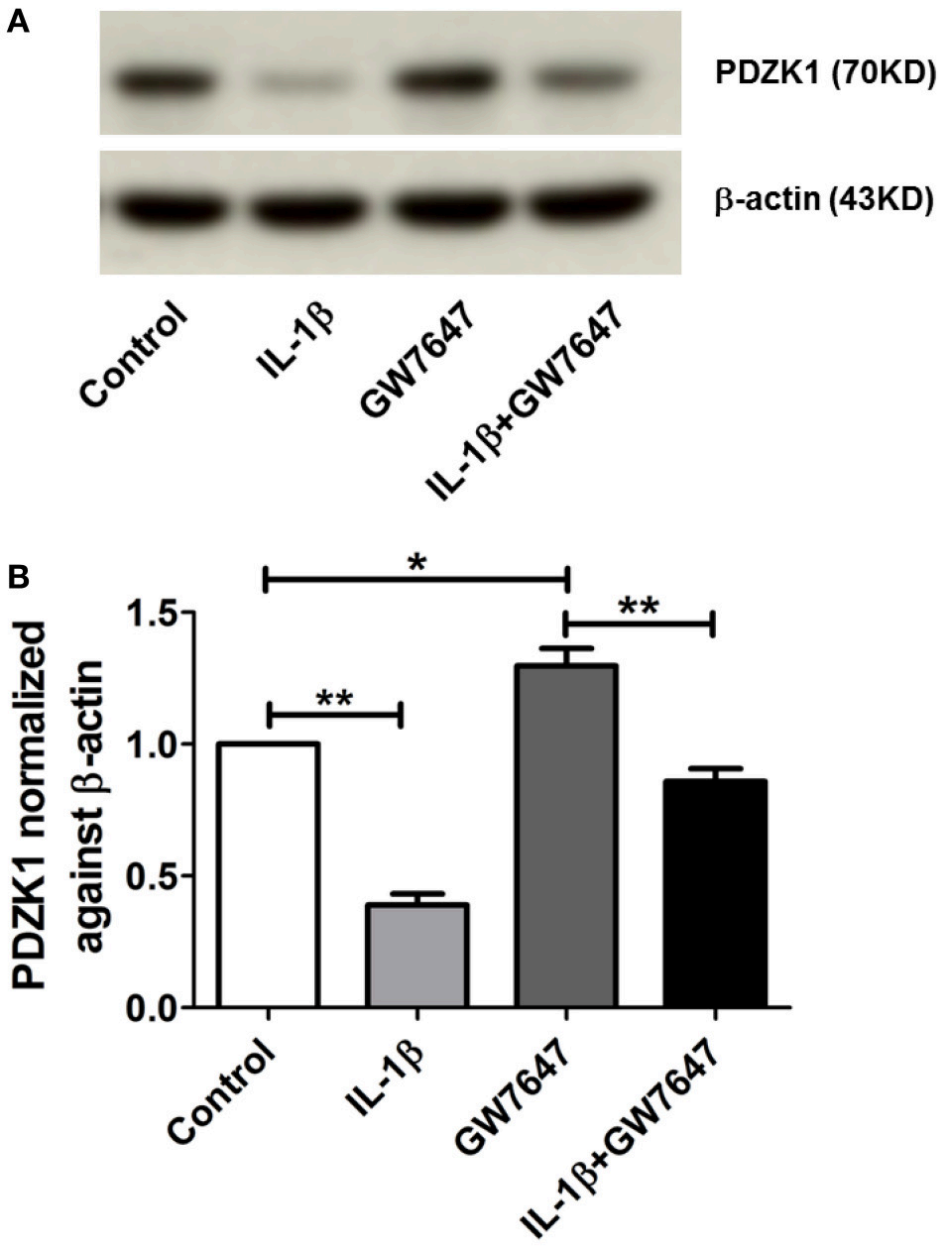
**Effects of PPARα agonist (GW7647) on PDZK1 protein expression. (A)** Caco-2BBE cells were pre-treated for 1 h with PPARα agonist GW7647 at a concentration of 1 μM and then treated with IL-1β (10 ng·mL^−1^) in Caco-2BBE cells for an additional 48 h. Cells were harvested after the treatment for protein isolation. Western blot analysis of PDZK1 protein expression and **(B)** Image J quantification of the blots normalized to β-actin expression. Treatment with GW7647 clearly abrogated the IL-1β mediated PDZK1 down regulation. Bar graphs are represented as mean ± SEM. ^*^indicates *P* < 0.05 and ^**^indicates *P* < 0.01. *n* = 3 experimental repeats, done in triplicates for each condition.

The transcription factor peroxisome proliferator-activated receptor alpha (PPARα) has been identified as a strong activator of the PDZK1 promoter (Tachibana et al., 2008). The peroxisome proliferator response element, to which the heterodimer PPARα and RXRα binds, is not located in the 5′ end of the PDZK1 promoter, but close to the start of transcription site at 98–86 bp, thus, not in the region sensitive to IL-1ß. Interestingly, IL-1β incubation also decreased PPARα expression in Caco2BBE cells to 65.5 ± 9.6% at 24 h incubation (Supplementary Figure 4), whereas a significant effect on PDZK1 mRNA expression was seen earlier (Figure 3A). It is nevertheless feasible that a PPARα agonist is able to restore PDZK1 expression in pro-inflammatory settings, offering a window for pharmacological intervention. We therefore incubated Caco2BBE cells with the PPARα agonist GW7647, in the absence and presence of IL-1β. At 24 h after GW7647 application, resulted in a significant increase of PDZK1 protein expression to 129.7 ± 6.5% of vehicle treated control (Figures 8A,B), and GW7647 was able to attenuate the IL-1β-mediated decrease in PDZK1 expression (61.1 ± 3.4% in the absence and 33.9 ± 2.3% in the presence of GW7647).

## Discussion

This study investigated the effect of three cytokines IL-1β, TNF-α, and IFN-γ on PDZK1 gene expression in Caco-2BBE cells, since these cytokines were known to be released in the mucosa of IBD patients (Reinecker et al., 1993; Dionne et al., 1997; Raddatz et al., 2005; Matsuda et al., 2009; Yeruva et al., 2010, 2015). We found that IL-1β strongly decreased PDZK1 promoter activity, mRNA and protein expression, whereas even high concentrations of the other two Th1 cytokines did not have a significant inhibitory effect. Since it is difficult to mimic the constant release of cytokines from immune cells in the lamina propria in an *in vitro* experimental setting, the experiments do not completely rule out a role for the tested, as well as other cytokines, on PDZK1 expression *in vivo*. Since we had found IL-1β expression 10-fold upregulated in the mucosa of patients with moderately active chronic ulcerative colitis under treatment compared to inactive colitis, whereas the other two cytokines were only ~2-fold upregulated in this chronic stage (Yeruva et al., 2010, 2015), IL-1β seemed a pathophysiologically meaningful target molecule for further investigation. PDZK1 was found strongly downregulated in the same mucosal tissues (Yeruva et al., 2015). IL-1β was also strongly upregulated and PDZK1 strongly downregulated in inflamed murine liver (Sharma, unpublished results), and indeed IL-1β had a similar effect on PDZK1 expression in hepatocytes as in enterocytes (Supplementary Figure 1). Therefore, IL-1β seemed a likely, although not necessarily an exclusive mediator of, inflammation-induced decrease in PDZK1 expression.

IL-1β exerted a relatively slow and surprisingly long-lasting effect on PDZK1 mRNA expression with a maximum effect 12 and 24 h after addition to the medium of the Caco-2BBE cells, and on PDZK1 protein expression at 24 and 48 h. Because approx. one third of IBD patients maintain mucosal inflammation and suffer from inflammatory diarrhea despite appropriate anti-inflammatory treatment, we asked the question whether the PDZK1 downregulation can be attenuated by inhibiting IL-1β receptor downstream signaling pathways. The IL-1β receptor was shown to influence gene expression by a number of signaling pathways, the best known being NF-κB, p38MAPK, JNK, and ERK1/2 cascades. In a previous study in Caco2 cells investigating IL-1β-stimulated chemokine expression, IL-1β was found to act majorly through NF-κB and p38MAPK pathways (Yeruva et al., 2008). Surprisingly, neither of these pathways seems responsible for the IL-1β mediated suppression of PDZK1. Inhibition of NF-κB signaling with BAY11-7082, which had been previously shown to inhibit IL-1β induced chemokine expression in Caco2 cells (Yeruva et al., 2008), did not significantly influence the downregulation of PDZK1 protein levels (Figure 5) or promoter activity by IL-1β (Supplementary Figure 3). Instead, we observed a decrease in the basal PDZK1 protein expression as well as promoter activity on BAY11-7082 treatment, suggesting that either NF-κB pathway is required for basal PDZK1 promoter activity or that BAY11-7082 exerted effects not directly related to NF-κB inhibition. Interestingly, a recent report has shown that the mode of action of BAY11-7082 involves inhibition of the ubiquitin conjugation machinery, leading to off-targets including the HIF-1α signaling pathway (Strickson et al., 2013). The fact that TNF-α stimulation resulted in a marked increase of NF-κB activity in Caco-2BBE cells (Musch et al., 2001; Carlson et al., 2007), but did not significantly decrease PDZK1 expression (this manuscript), corroborates the independence of IL-1β-mediated PDZK1 downregulation from NF-κB activation. While p38MAPK signaling is crucial for the pro-inflammatory cytokine gene-expression downstream to IL-1 signaling, the specific p38MAPK inhibitor BIRB-796 (Bain et al., 2007; Menon et al., 2011), did not attenuate the IL-1β-mediated decrease in PDZK1 protein expression or promoter activity (Figure 5). Moreover, a role for the stress-activated JNK-signaling was also ruled out, since the JNK1-3 inhibitor SP600125 neither affected basal expression nor rescued IL-1β-mediated downregulation of PDZK1. Interestingly, the MEK inhibitor PD98059, which inhibits the ERK1/2 (and ERK5) pathway, both increased basal PDZK1 expression in Caco-2BBE cells and diminished the relative decrease of PDZK1 expression by IL-1β (Figure 5D). To confirm the role of the ERK pathway signaling on PDZK1 expression we used U0126, another MEK inhibitor claimed to be specific for this pathway (Davies et al., 2000; Nishimoto and Nishida, 2006), and obtained similar effects (Figure 5F), suggesting that the ERK pathway negatively regulates PDZK1 expression. ERK activation inhibits differentiation in Caco-2BBE as well as other intestinal cell lines (Lemieux et al., 2011), and may do so in part by negatively regulating the expression of the differentiation marker PDZK1. While the results demonstrate that ERK inhibition at the level of the epithelial cells can reverse the negative effect of proinflammatory cytokine signaling on adaptor protein expression and thereby transport function, it is unlikely that a therapeutic strategy can be developed based on the inhibition of such a globally important signaling pathway. We therefore searched for potential targets for which inhibitor strategies are already in clinical use.

The region of the PDZK1 promoter between –4689 and –3995 bp was found to be important for both a major part of basal promoter activity as well as responsiveness to IL-1β. An *in silico* analysis of this region showed a variety of putative transcription factor binding elements, among them the retinoic acid response element (RARE; Supplementary Figure 2). Indeed, 9-cis retinoic acid (RA), a ligand for both nuclear receptors RAR and RXR, caused a rapid increase in PDZK1 mRNA expression within 3 h (Figure 7A), followed by an increase in PDZK1 protein expression (Figures 7B,C). In addition, RA and IL-1β co-incubation decreased the percentage of the IL-1β-induced decrease in PDZK1 mRNA and protein expression up to 48 h after addition (Figure 7). Since it has been previously shown that IL-1β decreases the expression of RXRα in liver cells (Li et al., 2002; Kim et al., 2007), and because RXRα was found to be downregulated in the liver during acute phase responses (Beigneux et al., 2000), we studied RXRα mRNA expression in colonic biopsies from UC patients and in Caco-2BBE cells after IL-1β treatment, and found a decrease in both (Figure 6). Interestingly, RXRα is a direct substrate of ERK1/2, and ERK-mediated phosphorylation suppresses its transactivation potential (Solomon et al., 1999), suggesting that both direct posttranslational modification of RXRα by ERK1/2 and downregulation of its expression by IL-1β, downstream signaling could contribute to the suppression of PDZK1 expression. Despite the inhibitory effect of IL-1β on RXRα-expression and possibly transactivation, RA was able to increase PDZK1 levels in the presence of IL-1β, opening a window for therapeutic intervention.

The PDZK1 promoter also contains a peroxisome proliferator response element (PPRE), to which PPARα binds as a heterodimer with RXRα (Tachibana et al., 2008). This response element is not in the part of the promoter that was most sensitive to IL-1β, but was close to the transcription start site (see Section Results). A reduced PPARα as well as PPARγ expression has been described in UC patient biopsies (Yamamoto-Furusho et al., 2011). Since the PPARα agonist GW7647 increased PDZK1 expression in Huh-7 hepatoma cells (Tachibana et al., 2008), as well as in Caco-2BBE cells (Figure 8), it is possible that the positive effect of RA on PDZK1 expression exerts its effect via binding of RXRα to the PPRE element as well as the RARE element. We wondered if the activation of the PPRE element by GW7647 may have a positive effect PDZK1 expression in the presence of IL-1β, and indeed GW7647 was also able to attenuate the inhibitory effect of IL-1β on PDZK1 expression.

PPARα dependent signaling pathways have been claimed to be involved both in the exacerbation (Qi et al., 2014) and the attenuation of colitis, the latter via enteric glia (Esposito et al., 2014). PPARγ also forms heterodimers with RXRα, and anti-inflammatory effects and amelioration of experimental colitis has been described with a number of PPARγ agonists through trans-repression of signal-dependent transcription factors that mediate inflammatory programs of gene activation (Linard et al., 2008). 5-ASA, the oldest and most widely used drugs in the treatment of inflammatory bowel diseases, is a PPAR agonist, but has many other inhibitory effects on inflammatory pathways (Perrotta et al., 2015). Many other PPAR agonists are in clinical use and novel ones are being tested.

In summary, IL-1β strongly downregulated PDZK1 expression in an ERK-dependent fashion in the intestinal cell line Caco-2BBE, demonstrating that the strong decrease in this important adaptor protein in inflamed intestine is a cytokine-mediated event and is attenuated by inhibiting MEK-dependent ERK activation. Stimulation of the nuclear receptors RXRα and PPARα increased PDZK1 expression, and attenuated the IL-1β induced downregulation. It may thus be possible to revert inflammation-induced epithelial damage and improve absorptive function even in the presence of ongoing cytokine release.

## Ethics statement

This study was carried out in accordance with the ethics committee of Hannover Medical School, with written informed consent from all subjects. All subjects gave written informed consent in accordance with the Declaration of Helsinki. The protocol was approved by the name of committee.

## Author contributions

Conception and design of the experiments: SY, MM, ML, and US; Collection, analysis, and interpretation of data: ML, SY, YL, GC, BR, KT, TD, US; Drafting the article SY, ML, and US; Revising it critically for important intellectual content: SY, MM, KT, TD, and US. All authors approved the final version of the manuscript.

### Conflict of interest statement

The authors declare that the research was conducted in the absence of any commercial or financial relationships that could be construed as a potential conflict of interest.

## Abbreviations

IL-1β: interleukin-1β
TNF-α: tumor necrosis factor-α
IFN-γ: interferon-γ
PDZ: PSD95, Disc-large and ZO1
PPAR α/γ: peroxisome proliferator-activated receptor α/γ
ERK: extracellular signal-regulated kinase
RXR α: retinoid X receptor α
JNK: c-Jun N-terminal kinase
NF-κB: nuclear factor-κB
MAPK: mitogen-activated protein kinase
TBP: TATA-box binding Protein
NHE3: Na^+^/H^+^ Exchanger 3
UC: ulcerative colitis.
