# Supplementary material for: IL-1β-Induced Downregulation of the Multifunctional PDZ Adaptor PDZK1 Is Attenuated by ERK Inhibition, RXRα, or PPARα Stimulation in Enterocytes
Source: Front Physiol. 2017 Feb 7;8:61. doi: 10.3389/fphys.2017.00061 (PMC5293818; doi:10.3389/fphys.2017.00061)
Supplement: Supplementary file 1 [file DataSheet2.pdf]

Supplementary Figures

S-Figure 1 Interleukin-1β decreased PDZK1 expression in Huh-7 cells

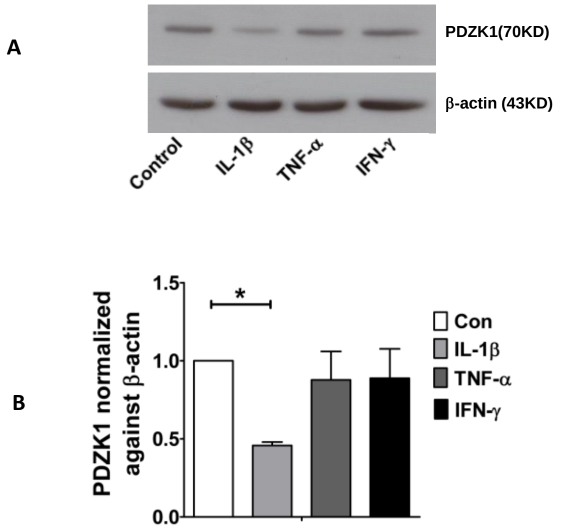

S-Figure 1. Interleukin-1β decreased PDZK1 expression in Huh-7 cells

A) Huh-7 cells (Hepatoma cells) were treated with IL-1β (10 ng·mL^{-1}), TNF-α (20 ng·mL^{-1}) and IFN-γ (30 ng·mL^{-1}) for 48 hours. Cells were harvested after the treatment for protein isolation. Huh-7 cells treated with IL-1β displayed PDZK1 downregulation, however, TNF-α and IFN-γ – treated Huh-7 cells did not. B) Image J quantification of the blots normalized to β-actin expression.Bar graphs are represented as mean ± SEM. n=3 experimental repeats done in different passages.

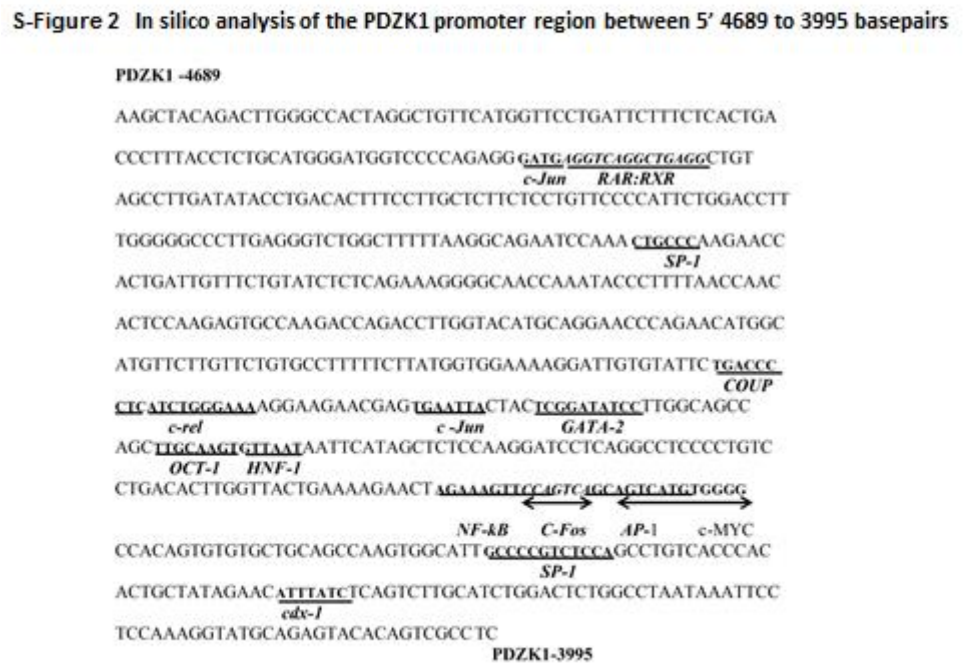

S-Figure 2. *In silico* analysis of the PDZK1 promoter region between 5' -4689 to -3995 basepairs.

PDZK1 5′-flanking sequence spanning 4689-3995 base pairs was subjected to transcription factor binding site prediction analysis with the TESS server (Transcription Element Search System). Putative binding sites for several transcription factors are found in this region, which are highlighted here.

S-Figure 3 Effects of NF-κB and p38-MAPK inhibitors on PDZK1 promotor activity

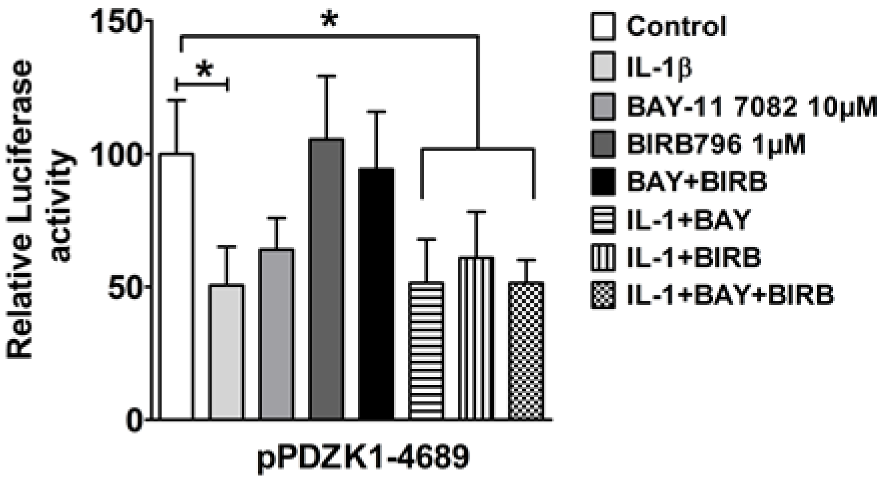

S-Figure 3. Effects of NF-κB and p38-MAPK inhibitors on PDZK1 promoter activity

Caco-2BBE transfected with a full length PDZK1 promoter-containing luciferase reporter plasmid (pPDZK1-4689) were pre-treated for 1 hour with the NF-κ inhibitor BAY11-7082 (10 μM) and/or the p38-MAPK inhibitor BIRB-796 (1 μM) before and during incubation with IL-1β (10 $ng \cdot mL^{-1}$). IL-1β displayed a strong inhibition of PDZK1 promoter activity, but blocking either NF-κB or p38-MAPK pathway, or both, did not significantly influence the inhibition of PDZK1 promoter activity by IL-1β. Bar graphs are represented as mean ± SEM. * indicates $p < 0.05$ compared to control. n=3 experimental repeats done in triplicates for each condition.

S-Figure 4: Suppression of PDZK1, RXRα and PPARα mRNA by IL-1β follow a different time course

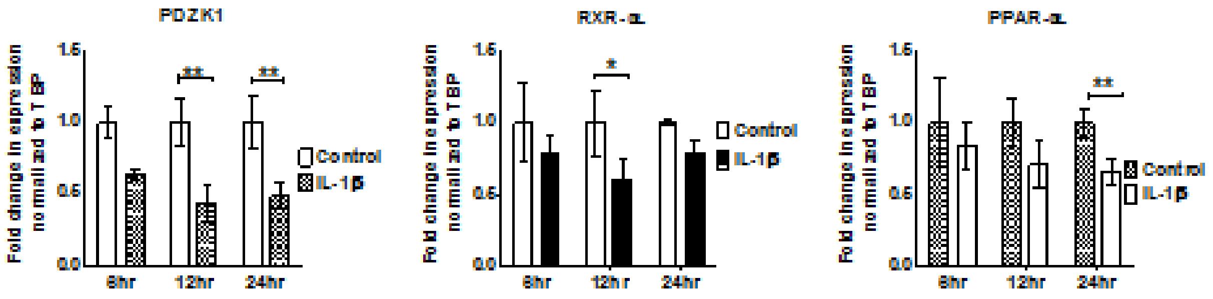

S-Figure 4 IL-1β decreased PPAR-α mRNA expression is time dependent in Caco2bbe cells

PPAR-α mRNA expression was analysed by real-time PCRs in Caco-2BBE cells after treatment with IL-1β (10 ng·mL^{-1}) for 6, 12 and 24 hours, data are represented as fold change in expression normalized to TBP. IL-1β decreased PPAR-α mRNA significantly by 24 hours. n=3 experimental repeats done in duplicates for each condition. Bar graphs represent mean ± SEM. * indicates $P < 0.05$ compared to control.

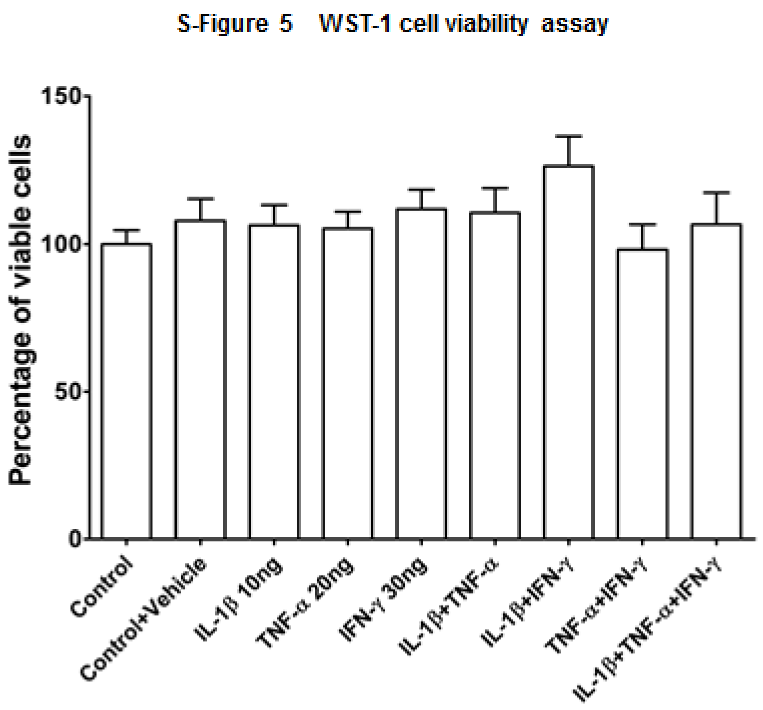

S-Figure 5. Effects of cytokine treatment on Caco-2BBe cell viability.

Caco-2BBe cells were treated as mentioned in material and methods, and WST-1 assays were performed to monitor cell viability during the cytokine incubation period. No cytotoxic effect was observed during the observation time. n=2 experimental repeats done in two different passages and in each experiment 8 wells were used for each condition.
